# Systematic review of antiepileptic drugs’ safety and effectiveness in feline epilepsy

**DOI:** 10.1186/s12917-018-1386-3

**Published:** 2018-03-02

**Authors:** Marios Charalambous, Akos Pakozdy, Sofie F. M. Bhatti, Holger A. Volk

**Affiliations:** 1 Small Animal Department, Faculty of Veterinary Medicine, Ghent University, Merelbeke, Belgium; 2 Clinical Unit of Internal Medicine Small Animals, University of Veterinary Medicine, Vienna, Austria; 3 Department of Clinical Science and Services, Royal Veterinary College, Hawkshead Lane, Brookmans Park, UK

**Keywords:** comprehensive review, epilepsy, feline, antiepileptic drugs, efficacy, adverse effects

## Abstract

**Background:**

Understanding the efficacy and safety profile of antiepileptic drugs (AEDs) in feline epilepsy is a crucial consideration for managing this important brain disease. However, there is a lack of information about the treatment of feline epilepsy and therefore a systematic review was constructed to assess current evidence for the AEDs’ efficacy and tolerability in cats. The methods and materials of our former systematic reviews in canine epilepsy were mostly mirrored for the current systematic review in cats. Databases of PubMed, CAB Direct and Google scholar were searched to detect peer-reviewed studies reporting efficacy and/or adverse effects of AEDs in cats. The studies were assessed with regards to their quality of evidence, i.e. study design, study population, diagnostic criteria and overall risk of bias and the outcome measures reported, i.e. prevalence and 95% confidence interval of the successful and affected population in each study and in total.

**Results:**

Forty studies describing clinical outcomes of AEDs’ efficacy and safety were included. Only two studies were classified as “blinded randomised controlled trials”. The majority of the studies offered high overall risk of bias and described low feline populations with unclear diagnostic criteria and short treatment or follow-up periods. Individual AED assessments of efficacy and safety profile showed that phenobarbital might currently be considered as the first choice AED followed by levetiracetam and imepitoin. Only imepitoin’s safety profile was supported by strong level of evidence. Imepitoin’s efficacy as well as remaining AEDs’ efficacy and safety profile were supported by weak level of evidence.

**Conclusions:**

This systematic review reflects an evidence-based assessment of the published data on the AEDs’ efficacy and safety for feline epilepsy. Currently, phenobarbital is likely to be the first-line for feline epileptic patients followed by levetiracetam and imepitoin. It is essential that clinicians evaluate both AEDs’ effectiveness and tolerability before tailoring AED to the individual patient. Further studies in feline epilepsy treatment are by far crucial in order to establish definite guidelines for AEDs’ efficacy and safety.

**Electronic supplementary material:**

The online version of this article (10.1186/s12917-018-1386-3) contains supplementary material, which is available to authorized users.

## Background

There is a paucity of literature about feline epilepsy compared to canine epilepsy, however epileptic seizures are a common neurological manifestation in cats with an estimated prevalence in a referral hospital population of 0.5-3.5% [1, 2]. Idiopathic epilepsy (IE) has not been studied in cats as thoroughly as in dogs but between 21-59% of cats presenting with recurrent seizures can be diagnosed with IE [3–5].

Treatment between canine and feline epileptic patients is rather different mainly due to the divergent safety profile between the two species [6, 7] but it is acceptable that in both species the effectiveness of an AED may outweigh its adverse effects, and therefore both the efficacy and safety profile should be considered before choosing the most appropriate AED(s) for patients [8].

Systematic reviews are powerful and objective tools to evaluate AEDs’ efficacy as well as severity and incidence of AEDs’ safety profile described in literature [9–12]. Guidelines with regards to AEDs’ efficacy and safety have been established in canine epilepsy [13, 14] based on previous systematic reviews and meta-analysis [8, 15]. However, such guidelines lack in feline epilepsy. The goal of this systematic review was to summarize and assess the results of the current studies regarding AEDs’ efficacy and safety in cats and provide evidence on the treatment of feline epilepsy.

## Methods

The methodology followed in this study was based on previous similar studies published by the authors [8, 15].

### Search strategy

Studies evaluating or describing the effectiveness and safety of an AED in cats were searched. As it was described in our previous systematic reviews [8, 15], studies were assessed based on four inclusion criteria adapted for feline patients, i.e. type of study (any peer-reviewed study), case definition (cats that were investigated and diagnosed with confirmed or presumed idiopathic epilepsy), treatment (cats treated with antiepileptic drugs only) and outcome (description of efficacy or safety outcomes after treatment). Final electronic searches were carried out on 21 June 2017 with no date or language restrictions and the same databases were searched with the same search strategies as it was described before [8, 15], i.e. Pub Med, CAB Abstracts, Google Scholar and searching of articles, proceedings and textbooks to identify reference lists of published papers and proceedings of relevant scientific conferences such as the annual American College of Veterinary Internal Medicine forum and the European College of Veterinary Neurology Symposium. The search terms used for the electronic databases were also the same as in our two previous systematic reviews on canine [8, 15] but adapted for feline subjects, i.e. the terms ‘dog’ and ‘canine’ were replaced by the terms ‘cat’ and ‘feline’, respectively.

### Study Selection

The same two-stage screening process that was recruited in our previous systematic reviews [8, 15] was used; in summary, at stage 1, papers’ titles and abstracts were only evaluated and at stage 2, full-length papers were evaluated based on the inclusion criteria 2, 3 and 4 to exclude the ones irrelevant to our outcomes.

### Assessment of quality of evidence

As in our previous systematic reviews [8, 15], studies were categorised based on their study design, i.e. “blinded randomised clinical trials” (bRCTs), “blinded randomised experimental laboratory animal studies” (bRELAS), “non-blinded RCTs” (nbRCTs), “non-blinded randomised ELAS” (nbRELAS), “non-randomised clinical trials” (NRCTs), “non-randomised ELAS” (NRELAS), “uncontrolled clinical trials” (UCTs), “uncontrolled ELAS” (UELAS), “case series and case reports [16, 17]”. In addition, the same three-part system of evidence quality evaluation to characterize weaknesses and strengths of each study within each group was used as it was described in our previous systematic reviews [8, 15], i.e. “(a) study group sizes, (b) subject enrolment quality and (c) overall risk of bias based on Cochrane [18] and Syrcle’s [19] ‘risk of bias’ assessment tool in order to provide an indicator of confidence associated with the findings of each study”. All in all, bRCTs or bRELAS with large population, clear and thorough diagnostic criteria and low overall risk of bias were considered as studies with the highest quality of evidence. Lastly, the treatment or follow-up period was reported for each study and was considered short or long if it was less or more than 6 months, respectively.

### Study group sizes

The same system that was reported in our previous systematic reviews [8, 15] was used to evaluate the population size in each study, i.e. “(a) >50 subjects per group (‘good’ number), (b) 20–50 subjects (‘moderate’ number), (c) 10–19 subjects (‘small’ number) and (d) <10 subjects (‘very small’ number)”.

### Assessment of subject enrolment quality

Information with regards to the investigations used for the diagnosis of IE were reviewed to assess the quality of subject definition in each study as ‘well characterized’, ‘fairly characterized’, ‘poorly characterized’ or ‘unclear.’ As far as the ELAS are concerned, that included investigations for describing non-epileptic healthy animals, two categories were described, i.e. ‘clearly characterized’ and ‘unclear’. The systems used were adapted for feline patients from our previous systematic reviews [8, 15]. The main difference is that for the “well characterized” category, tests for infectious diseases (including feline leukemia virus, feline immunodeficiency virus, feline infectious peritonitis, toxoplasmosis) were also included but these were desirable but not mandatory.

### Assessment of overall risk of bias and level of the studies’ evidence

The same methodology followed in our previous systematic review was used [8], i.e. “the studies were assessed based on the ‘risk of bias’ components and were categorised as presenting ‘high’, ‘low’ or ‘unclear’ risk for each component. The components were the random sequence generation, allocation concealment, blinding of participants, personnel and outcome assessment, completeness of outcome data, selective reporting of outcomes, random housing and baseline characteristics of cats (only for ELAS) and other sources of bias”.

### Assessment of outcome measures

The outcome measure was the assessment of the effectiveness and tolerability of AED(s) administered in cats.

### AEDs’ effectiveness

The assessment of the effectiveness was performed based on our previous canine systematic review [15]. In addition, the 95% confidence interval (CI) of the successful population in each study was also calculated and assessed as previously [15]; in summary, in each study, the cats with ≥50% reduction in seizure frequency were considered as successful cases and if the 95% CI of successfully treated cases was within ranges greater than 50%, then it was assumed that the majority of the study population was successfully treated.

### AEDs’ safety

The adverse effects were categorised by organ system, i.e. neurological, gastro-intestinal, etc. and types, i.e. type I (dose-dependent) and type II (idiosyncratic or dose-independent). The assessment of the safety was performed based on our previous systematic review [8]; in summary, the prevalence of the affected population in each study (i.e. number of cats that experienced adverse effects divided by the total size of the study population) and proportion of specific adverse effects for each individual AED (i.e. number of studies that reported a specific adverse effect divided by the total number of the studies for this AED) were evaluated. A further outcome measure was also added which was the proportion of specific adverse effects for each AED based on the total affected population (i.e. number of subjects that developed a specific adverse effect divided by the total population size from all the studies). In addition, the 95% CI of the affected population in each study was also calculated and assessed as previously [8]. If the 95% CI of the affected cases was within ranges greater than 50%, then it was considered that the majority of the study population showed adverse effects.

## Results

### Description of studies

By 21 June 2017, a total number of 684 unique citations were found; 676 articles and eight major conference abstracts from manual and electronic searches. One hundred seventy six items fulfilled stage 1 screening criteria, of which, 40 final studies (published between 1973 and 2017) also fulfilled stage 2 selection criteria and were therefore chosen for thorough evaluation. The process is also shown via a flow diagram (Additional file 1). One study included three different trials [20] and another study included both a trial and a retrospective part [21]. The included studies represented 2/40 (5%) bRELAS, 1/40 (2.5%) nbRCT, 2/40 (5%) nbRELAS, 5/40 (12.5%) UCTs, 11/40 (27.5%) UELAS, 12/40 (30%) retrospective case series and 7/40 (17.5%) case reports (Table 1). Overall, the 40 selected studies described 10 different AEDs. AEDs were usually given orally in all but three studies, where medication was given intraperitoneally or via transdermal application.

**Table 1:** Details of study design, risk of bias, disease characterization and study group size

	Study design	Risk of bias						Disease definitions (characterization)	Study groups size
		Blinding of outcome assessment	Randomization	allocation concealment	Incomplete outcome data	Selective reporting	other sources of bias		
Engel et al. [20]study 1	bRELAS	low	low	high	low	high	high; company funding	well	very small
Engel et al. [20] study 2		low	low	high	low	high	high; company funding	well	very small
Lowrie et al. [26]	nbRCT	high	low	high	high	low	unclear	poorly	good
Sawchuk et al. [47]	nbRELAS	high	low	low	low	high	unclear	clear	small
Carnes et al. [42]		high	low	high	low	high	unclear	clear	small
Engel et al. [20] study 3	UCT	high	high	high	low	high	high; company funding	well	very small
Dewey et al. [41]		high	high	high	low	high	high; conference abstract	unclear	very small
Ukai et al. [51]		high	high	high	high	high	unclear	well	very small
Volk et al. [36]		high	high	high	low	low	high; conference abstract	well	very small
Bailey et al. [40]		high	high	high	high	high	unclear	fairly	small
Roye et al. [48]	UELAS	high	high	high	low	high	unclear	unclear	very small
Barnard et al. [43]		high	high	high	low	high	unclear; conference abstract	clear	very small
Solomon et al. [34]		high	high	high	low	high	unclear	unclear	very small
Hasegawa et al. [50]		high	high	high	low	high	unclear	unclear	very small
Pellegrini et al. [53]		high	high	high	low	high	unclear	unclear	very small
Cochrane, Black et al. [32]		high	high	high	low	high	unclear	clear	very small
Cochrane, Parent et al. [33]		high	high	high	low	high	unclear	clear	very small
Boothe et al. [21]		high	high	high	low	high	low	clear	very small
Cautela et al. [52]		high	high	high	low	high	high; conference abstract	clear	very small
Gasper et al. [35]		high	high	high	high	high	unclear	clear	small
Dreimann [55]		high	high	high	low	high	High; abstract; dissertation	unclear	small
Schwartz-Porsche and Kaiser [44]	retrospective case series	NA						unclear	moderate
Brewer et al. [49]								unclear	very small
Center et al. [45]								unclear	small
Hughes et al. [46]								clear	very small
Wagner [38]								unclear	moderate
Boothe et al. [21]								unclear	small
Volk et al. [28]								well	small
Schriefl et al. [4]								fairly	small
Bertolani et al. [37]								unclear	very small
Pakozdy et al. [27]								fairly	moderate
Finnerty et al. [25]								well	small
Wahle et al. [31]								well	small
Ducote et al. [23]	Case reports	NA						NA	very small
Zoran et al. [54]								clear	very small
Lieser and Schwedes 2016								NA	very small
Boydell [29]								well	very small
Baho et al. [22]								NA	very small
Klang et al. [39]								well	very small
Cuff et al. [30]								well	very small

Sample size; >50 subjects per group (‘good’ number), 20–50 subjects (‘moderate’ number), 10–19 subjects (‘small’ number) and (d) <10 subjects (‘very small’ number)

### Signalment and baseline characteristics of study subjects

Baseline characteristics, i.e. breed, age and sex, of feline population were reported to some degree in all the studies. Signalment was reported in all the clinical studies, with various breeds, both sexes and a wide range of ages at study entry (range 0.25-19 years) being reported. The most common reported breed was domestic shorthaired cats followed by Birmans, domestic longhaired cats, Siamese, Burmese, Bengals, Himalayan and Maine Coon. Males were more commonly affected compared to females, though these results were not statistically examined in order to specifically report the prevalence of IE on the grounds of sex.

### Disease characterization and subject enrolment quality

In approximately half of the studies (19/40, 48%), the inclusion criteria for diagnosing IE (clinical studies) or healthy cats (ELAS) were not well described (Table 1). Three case reports, each one described an otherwise healthy cat that was suspected with structural epilepsy due to an anesthesia-related hypoxic event [22] or post-tramatic/vascular event [23, 24]. However, these studies were included in our review for evaluating the AED safety profile, as their aim was to describe an AED-related adverse effect that was unrelated to the cause of the epilepsy.

### Study group sizes

Many studies (36/40, 90%) assessed small or very small study size groups (Table 1).

### Methodological quality of included studies

Many studies (96%) showed high or unclear risk of bias (Table 1).

### AEDs efficacy and safety profile

**Fig. 1:**
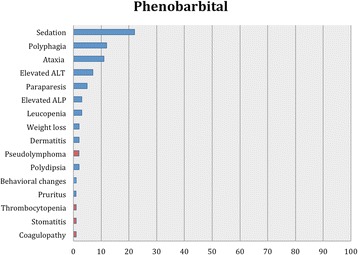
Proportion of specific adverse effects for phenobarbital. Each adverse effect represents the percentage of cats that were affected by this with regards to the overall combined population for phenobarbital. The blue and red bars indicate type I and II adverse effects, respectively

**Fig. 2:**
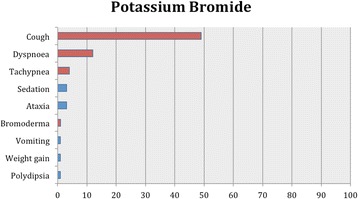
Proportion of specific adverse effects for potassium bromide. Each adverse effect represents the percentage of cats that were affected by this with regards to the overall combined population for potassium bromide. The blue and red bars indicate type I and II adverse effects, respectively

**Fig. 3:**
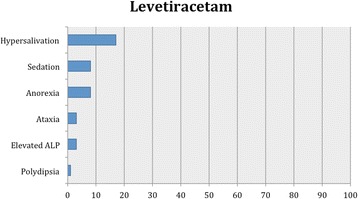
Proportion of adverse effects for levetiracetam. Each adverse effect represents the percentage of cats that were affected by this with regards to the overall combined population for levetiracetam. The blue bars indicate type I adverse effects

**Fig. 4:**
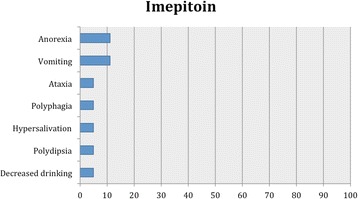
Proportion of specific adverse effects for imepitoin. Each adverse effect represents the percentage of cats that were affected by this with regards to the overall combined population for imepitoin. The blue bars indicate type I adverse effects

As in our previous systematic reviews [8, 15], details of studies’ data, characteristics and outcomes are summarized in the manuscript with further details captured in Additional file 2: Table S1, Tables 2, 3 and 4 and Figs. 1, 2, 3 and 4.

**Table 2:** Details of feline population size, seizure frequency, treatment time, doses of AED(s), seizure frequency reduction after AED initiation, 95% CI for the successful and affected cases and evidence statements for each study

References	Wagner [38]	Volk et al. [36]	Boothe et al. [21]	Boothe et al. [21] (retrospective part)	Bertolani et al. [37]	Volk et al. [28]	Klang et sl. [39]	Carnes et al. [42]	Bailey et al. [40]	Lowrie et al. [26]	Dewey et al. [41]	Cuff et al. [30]	Barnard et al. [43]
AED evaluated	Potassium Bromide							LEV					
2^nd^ AED	-	-	-	PB (12)	-	-	PB	-	PB (12)		PB (4)	PB	-
3^rd^ AED	-	-	-	-	-	-	LEV	-	-		-	-	
4^th^ AED	-	-	-	-	-	-	Gabapentin	-	-		-	-	-
No of cats	26	9	7	17	7	5	1	10 (non-epileptic cats)	12 (10 completed the efficacy analysis)	34 (28 completed the efficacy analysis)	4 (3 had presumed IE)	1	7 (non-epileptic cats)
Age of cats at seizure onset (years)	NA	mean 4.1	NA	mean 8.6	median 3; mean 3.2; range 1-8	NA	4	NA	median 2; range 0.25-16	median 16; range 10-19	NA	10	NA
Period of treatment or follow-up (months)	range 1.75-14	mean 13.63	2	mean 12.5	median 8; mean 18; range 1-96	NA	0.75	1 day	median 9; mean 12; range 6-24	3	Minimum 3 months	2	0.06
Dose of AED(s) (mg/kg)	NA	mean 16.5 PO BID	15 PO BID	12 PO BID	NA	NA	PBr 100 (total dose) PO BID; PB: 30 (total dose) PO BID; LEV: 50 (total dose) PO TID; gabapentin 100 (total dose) PO TID	20 mg/kg PO or IV SID (only one single dose)	PB: median 3.02; range 0.75-4.9 PO BID; LEV: median 23.6; range 17.2-34.7 PO TID	median 62.5; range 60-93.75 PO SID	20 mg/kg PO TID	PB: 4.5 PO BID; LEV 50 PO TID	500 mg PO single dose (extended release)
Serum levels of AED(s)	NA	mean 1.15 mg/ml	mean 1.1 mg/ml	PBr: mean 1.5 mg/ml. PB: range, 13-47 μg/ml	NA	NA	NA	median 26.77; mean 25.54; range 13.22-37.11 μg/ml	PB: median 29; range 5.6-75 μg/ml; LEV: median 25.5 μg/ml	NA	PB: mean 35.8 μg/ml; LEV: mean, 16.5; range: 6.9–24.3 μg/ml	NA	89.7 + /−25.7 μg/ ml
Pre-treatment SF (seizures/month or year)	NA	mean 4/month (recorder over a period of 6-12 months)	NA	NA	NA	NA	NA	NA	median 2.1; range 0.8-42.4/month (recorder over a period of median 4.5; range 0.3-46 months)	65/month	NA	continuous	NA
Post-treatment SF (seizures/month or year)	NA	0.52/month	NA	NA	NA	NA	NA	NA	median 0.42; range 0-1.25/month	NA	NA	0 (recorded over a period of 2 months)	NA
No of cats that were failures	NA	1/9 (11%)	NA	-	NA	0	NA	NA	0%	0/28 (0%)	1/4 (25%)	-	NA
No of cats with >0% - <50% reduction in SF	NA	-	NA	8/15 (53%)	NA	0	NA	NA	3/10 (30%)	0/28 (0%)	3/4 (75%)	-	NA
No of cats with ≥50% - <100% reduction in SF	NA	3/9 (33%)	NA	-	NA	0	NA	NA	4/10 (40%)	14/28 (50%)	0	-	NA
No of cats with 100% reduction in SF	NA	5/9 (56%)	NA	7/15 (47%) (available for 15 cats only and 3/7 were receiving also PB)	NA	5/5 (100%)	NA	NA	3/10 (30%)	14/28 (50%)	0	1/1 (100%)	NA
95% CI of successfully treated cases	NA	56-98%	NA	25-70%	NA	100%	NA	NA	40%-90%	100%	0%	100%	NA
Prevalence of adverse effects	11/26 (42%)	6/9 (67%)	0%	8/17 (47%)	NA	4/5 (80%)	NA	10/10 (100%)	2/12 (17%)	5/34 (18%)	2/4 (50%)	NA	0%
95% CI of cases that developed adverse effects	26-61%	35-88%	0%	26%-70%	NA	38-96%	NA	100%	4-45%	6-30%	15-85%	NA	0%
Body system affected and adverse effects	Respiratory (cough(11), dyspnea(2))	Respiratory (cough(6)), dermatological (dermatitis/bromoderma(1))	NA	Respiratory (cough (6)), GI (vomiting (1)), Neurological (sedation/ataxia (2)), weight gain (1), PD (1)	Respiratory (cough(7), dyspnea(2), tachypnea (2))	Respiratory (cough, dyspnea)	Respiratory (cough, tachypnea)	GI (hypersalivation)	Neurological (sedation(1)), GI (anorexia(2))	Neurological (sedation (4), ataxia (2), GI (anorexia (3)), PD (1)	ClinPath (increased ALP(2))	NA	NA
Most common adverse effects	Cough	Cough	NA	Cough	Cough	Cough	NA	hypersalivation (after PO administration only)	anorexia	sedation, anorexia	increased ALP	NA	NA
Adverse effect type	II	II	NA	I & II	II	II	II	I	I	I	I	NA	NA
Proportion of specific adverse effects for each AED based on all study reports	Type I: sedation, ataxia, vomiting, weight gain and PD (1/7; 14%)							Type I: sedation (2/5; 40%), anorexia (2/5; 40%), ataxia (1/5; 20%), hypersalivation (1/5; 20%), elevated serum ALP (1/5; 20%) and PD (1/5; 20%)					
	Type II: cough (6/7; 86%), dyspnea (3/7; 43%), tachypnea (2/7; 29%), dermatitis/bromoderma (1/7; 14%)												
	One study reported that there were no adverse effects.												
Proportion of specific adverse effects for each AED based on the total affected population	Cough (35/72; 49%), dyspnea (9/72; 12%), tachypnea (3/72; 4%), sedation (2/72; 3%), ataxia (2/72; 3%), dermatitis/bromoderma (1/72; 1%), vomiting (1/72; 1%), weight gain (1/72; 1%) and PD (1/72; 1%)							Hypersalivation (10/67; 15%), sedation (5/67; 7%), anorexia (5/67; 7%), ataxia (2/67; 3%), elevated serum ALP (2/67; 3%) and PD (1/67; 1%)					
Overall level of evidence supporting the efficacy and safety profile of an AED	Weak level of evidence for potassium bromide’s efficacy and safety profile							Weak level of evidence for levetiracetam’s efficacy and safety profile					

*AED(s)* anti-epileptic drug(s), *BID* bis in die (twice daily), *CI* confidence interval, *GI* gastrointestinal, *IE* idiopathic epilepsy, *LEV* Levetiracetam, *m* month(s), *NA* Not Available, *PB* phenobarbital, *PD* polydipsia, *PU* polyuria, *PP* polyphagia, *PBr* potassium bromide, *PO* per os, *SID* semel in die (once daily), *TID* ter in die (three times daily), *w* week(s), *y* year(s)

**Table 3:** Details of feline population size, seizure frequency, treatment time, doses of AED(s), seizure frequency reduction after AED initiation, 95% CI for the successful and affected cases and evidence statements for each study

References	Engel et al. [20] (ELAS 1)	Engel et al. [20] (ELAS 2)	Engel et al. [20] (Clinical trial)	Center et al. [45]	Hughes et al. [46]	Schwarz-Porsche & Kaiser, [44]	Sawchuk et al. [47]	Schwarz-Porsche & Kaiser, [44]	Roye et al., [48]	Schwarz-Porsche & Kaiser [44]
AED evaluated	Imepitoin			Diazepam			Primidone		Phenytoin	
2^nd^ AED	-					-	-	-	-	Diazepam
3^rd^ AED	-					-	-	-	-	-
4^th^ AED	-					-	-	-	-	-
No of cats	6	6	8 (7 were evaluated for the safety profile)	11 (non-epileptic cats)	5 (non-epileptic cats)	NA	11	6	4 (non-epileptic cats)	2
Age of cats at seizure onset (years)	NA	NA	median 4; mean 6.3; range 1-15	NA	range 1-12	range 0.2-9	NA	range 0.2-9	NA	range 0.2-9
Period of treatment or follow-up (months)	1	1	at least 2 (for the seizure-freedom cases the period was mean 4; range 2-7.5)	0.5	0.25-0.5	6	3	range 5-108	0.8	>3-9
Dose of AED(s) (mg/kg)	30 PO BID	40 & 80 PO BID	median 30; mean 27.92 PO BID	1.25-2 PO SID or BID	0.23-0.82 PO SID	0.5-2 PO (divided in 3 daily doses)	20 mg/kg PO BID	40-50 PO (divided in three daily doses)	10 PO and intramuscularly SID	1.5 PO SID
Serum levels of AED(s)	NA	NA	NA	NA	NA	NA	4.1 μg/mL	NA	25-35 μg/mL	6.5-17 μg/mL
Pre-treatment SF (seizures/month or year)	NA	NA	Median 20; mean 57.71; range 2-200		NA	NA	NA	NA	NA	NA
Post-treatment SF (seizures/month or year)	NA	NA	Median 1.5; mean 19.43; range 0-100	NA	NA	NA	NA	NA	NA	NA
No of cats that were failures	NA	NA	3/8 (37%)	NA	NA	-	NA	2/6 (33%)	NA	1/2 (50%)
No of cats with >0% - <50% reduction in SF	NA	NA	-	NA	NA	20%	NA	-	NA	-
No of cats with ≥50% - <100% reduction in SF	NA	NA	1/8 (13%)	NA	NA	40%	NA	2/6 (33%)	NA	-
No of cats with 100% reduction in SF	NA	NA	4/8 (50%)	NA	NA	40%	NA	2/6 (33%)	NA	1/2 (50%)
95% CI of successfully treated cases	NA	NA	30-86%	NA	NA	NA	NA	30-90%	NA	9-90%
Prevalence of adverse effects	0 % (apart from intermittent/rare vomiting)	0 % (apart from intermittent/rare vomiting, hypersalivation and slightly decreased appetite)	5/7 (71%)	NA	NA	0%	11/11 (100%)	1/6 (17%)	4/4 (100%)	2/2 (100%)
95% CI of cases that developed adverse effects	NA	NA	36-92%	NA	NA	0%	100%	3-56%	100%	100%
Body system affected and adverse effects	NA	NA	Neurological (sedation (2), ataxia (1)), GI (anorexia (2), PP (1), vomit (2), hypersalivation (1)), PD (1), decreased drinking (1)	Neurological (sedation(5), ataxia(5)), GI (acute hepatic necrosis(11), anorexia (5))	GI (acute hepatic necrosis)	NA	Neurological (sedation, ataxia)	Neurological (sedation), GI (anorexia, weight loss)	Neurological (sedation, ataxia) GI (anorexia)	GI (anorexia), ClinPath (increased liver enzymes)
Most common adverse effects	Intermitent vomit	Intermittent vomit	Sedation, vomit, decreased appetite	NA	NA	NA	NA	NA	NA	NA
Adverse effect type	I	I	I	I & II	II	NA	I	NA	I	I
Proportion of specific adverse effects for each AED based on all study reports	Type I: sedation (1/3; 33%) and ataxia (1/3; 33%), GI signs, i.e. anorexia (1/3; 33%), PP (1/3; 33%), vomiting (1/3; 33%), hypersalivation (1/3; 33%), and PD (1/3; 33%) or decreased water consumption (1/3; 33%)			Type I: sedation and ataxia			Type I: sedation (2/2; 100%), ataxia (1/2; 50%), anorexia (1/2; 50%) and weight loss (1/2; 50%)		Type I: anorexia (2/2; 100%), sedation (1/2; 50%), ataxia (1/2; 50%), increased liver enzymes (1/2; 50%)	
				Type II: acute hepatic necrosis						
				One study reported no adverse effects						
Proportion of specific adverse effects for each AED based on the total affected population	Sedation (2/19; 11%), anorexia (2/19; 11%), vomiting (2/19; 11%), ataxia (1/19; 5%), PP (1/19; 5%), hypersalivation (1/19; 5%), PD (1/19; 5%) and decreased drinking (1/19; 5%)			NA			Sedation (12/17; 71%), ataxia (11/17; 65%), anorexia (1/17; 6%) and weight loss (1/17; 6%)		Anorexia (6/6; 100%), sedation (4/6; 67%) ataxia (4/6; 67%) and increased liver enzymes (2/6; 33%)	
	Weak and good level of evidence for imepitoin’s efficacy and safety profile, respectively			Weak level of evidence for diazepam’s efficacy and safety profile			Weak level of evidence for primidone’s efficacy and safety profile		Weak level of evidence for phenytoin’s efficacy and safety profile	

AED(s), anti-epileptic drug(s); BID, bis in die (twice daily); CI, confidence interval; GI, gastrointestinal; IE, idiopathic epilepsy; LEV, Levetiracetam; m, month(s); NA, Not Available; PB, phenobarbital; PD, polydipsia; PU, polyuria; PP, polyphagia; PBr, potassium bromide; PO, per os; SID, semel in die (once daily); TID, ter in die (three times daily); w, week(s); year(s); y

**Table 4:** Details of feline population size, seizure frequency, treatment time, doses of AED(s), seizure frequency reduction after AED initiation, 95% CI for the successful and affected cases and evidence statements for each study

References	Hasegawa et al. [50]	Brewer et al. [49]	Ukai et al. [51]	Cautela et al. [52]	Pellegrini et al., [53]	Zoran et al., [54]	Dreimann [55]
AED evaluated	Zonisamide			Pregabalin	Valproic acid		
2^nd^ AED	-	PB (5)	-		-	-	-
3^rd^ AED	-	-	-		-	-	-
4^th^ AED	-	-	-		-	-	-
No of cats	6 (non-epileptic cats)	5	8	6 (non-epileptic cats)	8 (non-epileptic cats)	1 (non-epileptic cat)	6 (non-epileptic cats)
Age of cats at seizure onset (years)	NA	NA	median 8.75; range 6.75 -11.5	NA	NA	NA	NA
Period of treatment or follow-up (months)	2.1	3	0.75	NA	0.12-0.25	NA	0.5
Dose of AED(s) (mg/kg)	20 PO SID	mean 11.54; range 6.14-17 PO SID	2.5 PO BID for a week, then 5 PO BID	4 PO (one dose only)	range 25- 130 intraperitoneally TID	< 111 PO at once	40 PO and IV BID
Serum levels of AED(s)	median 52.9; mean 56.9 μg/mL	Zonisamide: mean 19.44; range 8.8-38.6 mcg/ml; PB: NA	median 5.9 (2.1–8.3) for a week, then 13.45 (11.9–24.4)	range 2.8-8.2 μg/mL	<5 μg/ml	3.4 μg/ml	50-150 μg/ml
Pre-treatment SF (seizures/month or year)	NA	NA	NA	NA	NA	NA	NA
Post-treatment SF (seizures/month or year)	NA	NA	NA	NA	NA	NA	NA
No of cats that were failures	NA	0	NA	NA	NA	NA	NA
No of cats with >0% - <50% reduction in SF	NA	2/5 (40%)	NA	NA	NA	NA	NA
No of cats with ≥50% - <100% reduction in SF	NA	3/5 (60%)	NA	NA	NA	NA	NA
No of cats with 100% reduction in SF	NA	0	NA	NA	NA	NA	NA
95% CI of successfully treated cases	NA	23%-88%	NA	NA	NA	NA	NA
Prevalence of adverse effects	3/6 (50%)	2/5 (40%)	0%	4/6 (67%)	>4/8 (>50%)	NA	6/6 (100%)
95% CI of cases that developed adverse effects	19-81%	12%-77%	0%	30-90%	NA	NA	100%
Body system affected and adverse effects	Neurological (sedation, ataxia) GI (vomiting, diarrhea, anorexia)	Neurological (sedation (1)), GI (anorexia (1))	0%	Neurological (sedation)	Neurological (sedation, ataxia, drowsiness, head tremor), GI (anorexia)	Neurological (hyperactivity), dermatological (alopecia)	Neurological (sedation), GI (vomiting, anorexia)
Most common adverse effects	NA	NA	NA	NA	Sedation, drowsiness, head tremor, anorexia	NA	NA
Adverse effect type	I	I		I	I	I & II	I
Proportion of specific adverse effects for each AED based on all study reports	Type I: sedation (2/3; 67%), anorexia (2/3; 67%), ataxia (1/3; 33%), vomiting (1/3; 33%) and diarhoea (1/3; 33%)			Type I: sedation	Type I: sedation (2/3; 67%), anorexia (2/3; 67%), ataxia (1/3; 33%), drowsiness (1/3; 33%), head tremor (1/3; 33%), hyperactivity (1/3; 33%) and vomiting (1/3; 33%)		
					Type II: alopecia (1/3; 33%)		
Proportion of specific adverse effects for each AED based on the total affected population	Sedation (4/19; 21%), anorexia (4/19; 21%), ataxia (3/19; 16%), vomiting (3/19; 16%) and diarrhea (3/19; 16%)			NA	Sedation (>10/15; 67%), anorexia (>10/15; 67%), ataxia (>4/15; 27%), drowsiness (>4/15; 17%), head tremor (>4/15; 17%), vomiting (6/15; 40%), hyperactivity (1/15; 7%) and alopecia (1/15; 7%)		
Overall level of evidence supporting the efficacy and safety profile of an AED	Weak level of evidence for zonisamide’s efficacy and safety profile			Absent and weak level of evidence for pregabalin’s efficacy and safety profile	Absent and weak evidence for valproic acid’s safety profile and efficacy respectively		

AED(s), anti-epileptic drug(s); BID, bis in die (twice daily); CI, confidence interval; GI, gastrointestinal; IE, idiopathic epilepsy; LEV, Levetiracetam; m, month(s); NA, Not Available; PB, phenobarbital; PD, polydipsia; PU, polyuria; PP, polyphagia; PBr, potassium bromide; PO, per os; SID, semel in die (once daily); TID, ter in die (three times daily); w, week(s); year(s); y

### Phenobarbital

Eight studies [4, 25–31] assessed the efficacy of phenobarbital as a monotherapy agent or in combination with other AEDs (two studies), providing a total size of 137 cats. Based on the 95% CI, in all the studies but three [4, 26, 31] (63%), the majority of the study population was treated successfully. In one of these studies though [26], phenobarbital was only used to treat a specific type of epileptic seizures in cats, i.e. feline auditory reactive seizures (FARS), which might have biased the results for the drug’s overall efficacy to control seizures.

Twelve studies [22–28, 31–35] evaluated the safety profile of phenobarbital, providing a total size of 147 cats. Three studies documented type I adverse effects, including dermatological and neurological signs, clinic-pathological abnormalities, polyphagia [36], polydipsia (PD) and weight loss (Additional file 2: Table S1). Four studies reported type II adverse effects including clinic-pathological abnormalities and lympho-reticular and gastrointestinal signs (GI) signs (Additional file 2: Table S1). Three studies reported that there were no adverse effects. Based on the 95% CI, in all studies but two [27, 34] (83%), the majority of reported population did not show adverse effects. The most common adverse effect was sedation and the least common were behavioral changes, pruritus, thrombocytopenia, ulcerative stomatitis and coagulopathy (Additional file 2: Table S1, Fig. 1).

The treatment or follow-up period was reported adequately in 14/15 (93%) studies (Additional file 2: Table S1). From these, in 9/14 (60%) the follow-up time was short (<6 months). Dose and serum levels were provided adequately in 13/15 (87%) and 11/15 (73%) studies respectively (Additional file 2: Table S1). The phenobarbital maintenance doses and serum levels were higher than the normal reference range [6] in 8/13 (62%) and 4/11 (36%) studies respectively. In all, type I and II adverse effects did not follow a dose or serum levels dependent pattern. In summary, the level of evidence for phenobarbital’s efficacy and safety profile was weak.

### Potassium Bromide

Three studies [21, 28, 36] assessed potassium bromide’s efficacy as a monotherapy agent or in combination to phenobarbital (one study), providing a total group size of 29 cats. Based on the 95% CI, in all the studies but one [21] (66%), the majority of the study population was treated successfully.

Six studies [21, 28, 36–39] evaluated the safety profile of potassium bromide, providing a total size of 72 cats. One of these studies included both a UCT and retrospective case series part [21]. One study documented type I adverse effects, such as neurological, GI signs and PD. Five studies reported type II adverse effects, such as respiratory and dermatological signs (Table 2). One study reported that there were no adverse effects. Applying the 95% CI, in all the studies (100%), the majority of the cases reported did not have adverse effects. The most common adverse effect was cough and least common were dermatitis/bromoderma, vomiting, weight gain and PD (Table 2, Fig. 2).

Treatment or follow-up time was reported adequately in 6/7 (86%) (Table 2). From these, in 2/6 (33%) the period was short (<6 months). The dose and serum levels were documented adequately in 4/7 (57%) and 3/7 (43%) studies (Table 2). There was no adequate information to relate specific range values of dose and serum levels with the development of type I or II adverse effects. In summary, the level of evidence for potassium bromide’s efficacy and safety profile was weak.

### Levetiracetam

Four studies [26, 30, 40, 41] assessed the efficacy of levetiracetam as a monotherapy agent or in combination to phenobarbital (three studies), providing a total group size of 43 cats. Based on the 95% CI, in two of the studies [26, 30] (50%), the majority of the study population was treated successfully.

Five studies [26, 40–43] reported the safety profile of levetiracetam, providing a total group size of 67 cats. Only type I adverse effects were reported in all evaluated studies, including neurological and GI signs and clinico-pathological abnormalities (Table 2). Based on the 95% CI, in all of the studies but one [42] (75%), the majority had no adverse effects reported. One study reported no adverse effects [43]. The most common adverse effect was hypersalivation and the least common was PD (Table 2, Fig. 3).

The treatment or follow-up time was reported adequately in all the studies (Table 2), but was short (<6 months) in 5/6 (83%). The dose and serum levels were reported adequately in 6/6 (100%) and 4/6 (66%) studies respectively (Table 2). The levetiracetam maintenance doses reported were higher than normal in 2/6 (33%) studies. In all, type I adverse effects did not follow a dose or serum levels dependent pattern. The level of evidence for levetiracetam’s efficacy and safety profile was weak.

### Imepitoin

One study [20] assessed the efficacy of imepitoin as monotherapy agent in 8 cats. With such a small population size studied the 95% CI could not demonstrate that the majority of cases were managed successfully.

Three studies, which were included as part of one report [20], reported the safety profile of imepitoin, providing a total study size of 19 cats. In one study, only type I adverse effects were reported including neurological, GI signs and PD or decreased water consumption. In the two other studies though, no such effects were reported but intermittent vomiting, hypersalivation and slightly decreased appetite (Table 3). Applying the 95% CI to all study data (100%), the majority of cases did not experience adverse effects. There was an equal distribution among all the adverse effects (Table 3, Fig. 4).

The treatment or follow-up time was reported adequately in all studies (Table 3). In all studies (100%) the period was short (<6 months). The dose and serum levels was provided adequately in 3/3 (100%) and 0/3 (0%) studies, respectively (Table 3). Higher imepitoin maintenance doses, i.e. 40 or 80 mg/kg PO BID, were associated with higher incidence of adverse effects. The level of evidence for imepitoin’s efficacy was weak but the level of evidence for safety profile was strong.

### Diazepam

One study [44] assessed the efficacy of diazepam as monotherapy. The study reported that the majority of the cases was managed successfully, but the 95% CI could not be calculated.

Three studies [44–46] reported the safety profile of diazepam, providing a total size of 16 cats. One study documented type I adverse effects, i.e. neurological signs (Table 3). Two studies documented type II adverse effects, i.e. GI signs (Table 3). One study reported that there were no adverse effects. Since all the studies, but one [44], selectively included cats that manifested adverse effects, the 95% CI and adverse effects prevalence could not be calculated (Table 3).

The treatment or follow-up time was reported adequately in 3/3 (100%), but was considered short in all (Table 3). The dose and serum levels were reported adequately in 3/3 (100%) and 0/3 (0%) studies respectively (Table 3). In all, type I and II adverse effects did not follow a dose or serum levels dependent pattern. The level of evidence for diazepam’s efficacy and safety profile was weak.

### Primidone

One study [44] assessed the efficacy of primidone as a monotherapy agent in 6 cats. Applying the 95% CI to such a small study population showed that the majority was not managed successfully.

Two studies [44, 47] reported the safety profile of primidone, providing a total group size of 17 cats. The studies reported type I adverse effects, such as GI and neurological signs and weight loss. No type II adverse effects were reported (Table 3). Applying the 95% CI to one study [44] (50%), the majority of cases did not have adverse effects reported. The most common adverse effect was sedation and the least common were anorexia and weight loss (Table 3).

The treatment or follow-up period was reported adequately in 2/2 (100%) (Table 3). From these, in 1/2 (50%) the period was short (<6 months). The dose and serum levels was reported adequately in 2/2 (100%) and 1/2 (50%) studies respectively (Table 3). There was inadequate information to relate specific range values of dose and serum levels with the development of type I or II adverse effects. The level of evidence for primidone’s efficacy and safety profile was weak.

### Phenytoin

One study [44] evaluated the efficacy of phenytoin as an adjunct to diazepam in 2 cats.

Two studies [44, 48] reported the safety profile of phenytoin, providing a total size of 6 cats. The studies documented type I adverse effects, such as GI and neurological signs and clinic-pathological abnormalities. No type II adverse effects were documented (Table 3). Based on the 95% CI, in all the studies (100%), the majority of the study population experienced adverse effects. The most common adverse effect was anorexia and the least common was increased serum liver enzymes (Table 3).

The treatment or follow-up period was reported adequately in 2/2 (100%) (Table 3). From these, in 1/2 (50%) the period was short (<6 months). The dose and serum levels were reported adequately in 2/2 (100%) studies (Table 3). There was inadequate information to relate specific range values of dose and serum levels with the development of type I or II adverse effects. The level of evidence for phenytoin’s efficacy and safety profile was weak.

### Zonisamide

One study [49] assessed the efficacy of zonisamide as an adjunct to phenobarbital in 5 cats. Applying the 95% CI to such a small study population showed that the majority was not managed successfully.

Three studies [49–51] reported the safety profile of zonisamide in 19 cats. The studies documented type I adverse effects, such as GI and neurological signs (Table 4). Based on the 95% CI, in all studies (100%), the majority of the study population did not have adverse effects. One study reported no adverse effects [51]

The treatment or follow-up time was reported adequately but was considered short in all studies (Table 4). The dose and serum levels were also reported adequately in all studies (Table 4). There was inadequate information to relate specific range values of dose and serum levels with the development of type I or II adverse effects. In summary, the level of evidence for zonisamide’s efficacy and safety profile was weak.

### Pregabalin

There were no original studies evaluating the efficacy of pregabalin in cats except for experts’ opinion. One study [52] reported the safety profile of pregabalin in 6 cats. The study documented type I adverse effects, i.e. neurological signs. No type II adverse effects were documented (Table 4). Applying the 95% CI to the studied population, the majority did not experience adverse effects.

The treatment or follow-up time was inadequately reported (Table 4). The dose and serum levels was reported adequately (Table 4). There was inadequate information to relate specific range values of dose and serum levels with the development of type I or II adverse effects. There was no evidence for pregabalin’s efficacy and an overall weak level of evidence for pregabalin’s safety profile.

### Valproic acid

There were no original studies evaluating the efficacy of valproic acid in cats except for experts’ opinion. Three studies [53–55] reported the safety profile of valproic acid, giving a combined sample size of 15 cats. Three studies documented type I adverse effects, such as GI and neurological signs. One study documented type II adverse effects, i.e. dermatological signs (Table 4). There was inadequate information to calculate the 95% CI but one study [55] showed that the whole population had adverse effects. The most common adverse effects were sedation ataxia, drowsiness, head tremor and anorexia and the least common were hyperactivity and alopecia.

The treatment or follow-up time was reported adequately in 2/3 (67%) studies and was considered short. The dose and serum levels were reported adequately in 3/3 (100%) (Table 4). There was inadequate information to relate specific range values of dose and serum levels with the development of type I or II adverse effects. There was no evidence for valproic acid’s efficacy and weak level of evidence for safety profile.

## Discussion

**Fig. 5:**
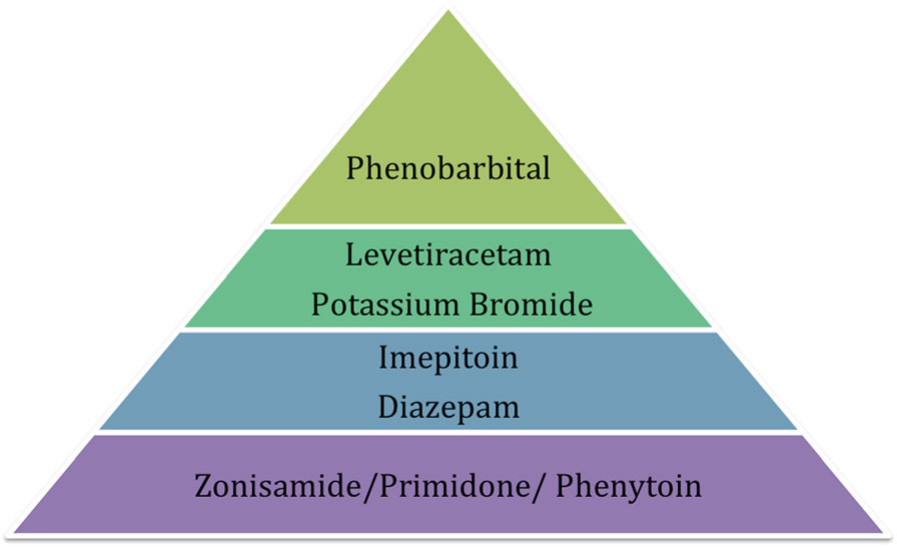
Pyramid of AEDs’ efficacy hierarchy based on the quality of evidence and outcomes assessment

**Fig. 6:**
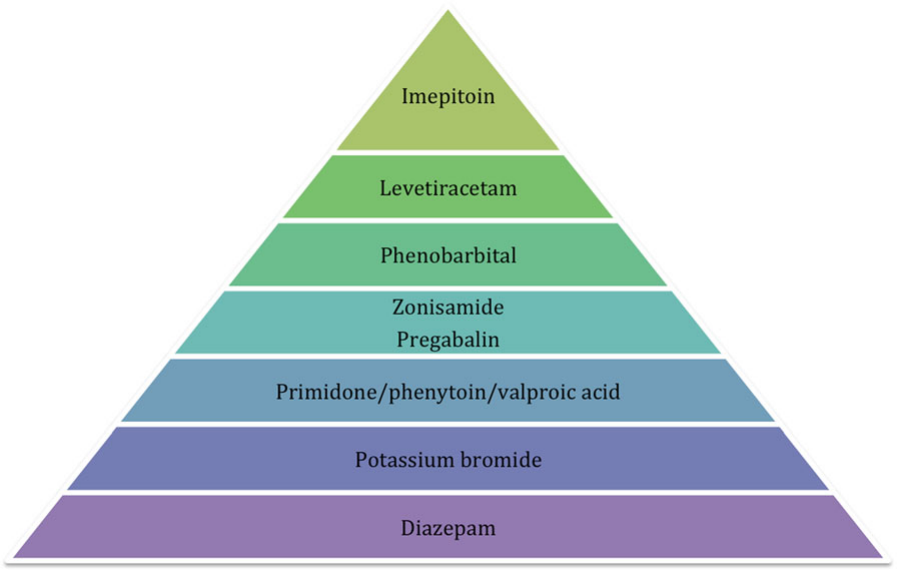
Pyramid of AEDs’ safety hierarchy based on the quality of evidence and outcomes assessment

To the authors’ knowledge, this is the first systematic review of AEDs’ efficacy and safety in cats. The authors were based on the PRISMA (Preferred Reporting Items for Systematic Reviews and Meta-Analyses) statement to report this systematic review [56]. The systematic review found that the level of evidence in feline epilepsy treatment is weak to absent, in particular for AEDs’ efficacy. The results showed that phenobarbital was considered the most effective AED followed by levetiracetam and potassium bromide, then imepitoin and diazepam and lastly zonisamide, primidone and phenytoin (Fig. 5). All supported by weak level of evidence. There was insufficient evidence for the efficacy of pregabalin and valproic acid. As far as the safety profile of all the AEDs is concerned, imepitoin was considered the safest AED, followed by levetiracetam and phenobarbital, then zonisamide and pregabalin followed by primidone, phenytoin and valproic acid and lastly potassium bromide and diazepam (Fig. 6). All supported by weak level of evidence apart from imepitoin, which was supported by good level of evidence. Although this systematic review focused on evaluating the use of AEDs in feline patients, it would be an omission not to mention that other substances have been proposed for managing seizures in cats [57, 58], with taurine as the most representative example. In a feline case report, administration of 300 mg taurine subcutaneously twice a day for two days followed by 100 mg taurine orally once a day for one month resulted in reduction in seizure frequency, supported by clinical and electroencephalographic observations. Abrupt cessation led to a rise of frequency [57]. Further clinical studies are crucial to support taurine’s potential efficacy in feline epilepsy.

The AEDs’ adverse effects were categorised as type I (predictable or dose-dependent) or type II (idiosyncratic/unpredictable or dose-independent) which are usually considered as non-immunological reactions [59]. It was challenging to detect a correlation between the AED dose or serum concentration and the type of adverse effects in feline patients. This could be attributed either to the possibility that, in feline patients, adverse effects might not follow a dose or serum related pattern or to the fact that there was inadequate information to allow us assessing any potential correlation between the incidence of adverse effects and the AEDs’ dose or serum concentrations. Severe type II adverse effects were reported mainly in cats being treated with potassium bromide or diazepam. Type II adverse effects are non-dose dependent, unpredictable, usually rare and caused by a cytotoxin, a drug or its metabolite [59]. Idiosyncratic reactions are most likely secondary to an individual difference in rate of formation and detoxification of reactive metabolites [59]. However, in the case of potassium bromide and, in particular, diazepam in feline patients, the incidence of these type II effects was high and in case of diazepam, they were considered life-threatening. This can give rise to evidence that the type II adverse effects of these two drugs in cats are species-related rather than individual-related.

In canine epilepsy, the majority of studies lacked a high level of evidence [8, 15]. However, in feline epilepsy, almost none of the studies provided high quality of evidence. The studies manifested high overall risk of bias. In addition, only 47% and 2% of all studies had well clinically defined groups and assessed a sufficient number of cats, respectively. The 95% CI, that was used as an indicator of the ‘real’ population of successful (AED efficacy) or affected (AED safety) cases, revealed a wide range of values mainly due to the inadequate feline population in the studies. Therefore, conclusions drawn based on the 95% CI results should be interpreted with caution. In addition, there were no bRCTs evaluating any AEDs in cats but two bRELAS assessing imepitoin’s safety profile. The follow up time was rather limited and short (<6months) to assess adequately long-term efficacy and tolerability. In all, due to the lack of studies with overall low risk of bias, insufficient disease characterisations and small group sizes, clear suggestions concerning AEDs’ efficacy and safety are difficult.

In contrast to canine epilepsy, many studies evaluated the efficacy and safety of an AED as monotherapy (with the exception of levetiracetam), making it easier to determine whether an AED’s adverse effects and efficacy were attributed mainly to its administration. However, certain evaluated factors might have influenced our assessment of AEDs’ efficacy and safety profile similar to the ones described in our previous systematic reviews for canine patients [8, 15]. These included the dosages used of a particular AED (i.e. different among studies), the frequency of AEDs’ administration (i.e. factor that could alter the chances of occurrence of adverse effects particularly in AEDs with short half-life due to fluctuations of serum drug levels; although this characteristic might not affect the results [60]), the duration of the study (i.e. insufficient duration, as it was found in many studies, might have reduced the chance for the most frequent adverse effects to occur or for adequately evaluate an AED’s efficacy). Lastly, a few AEDs have been used more often in feline epilepsy and been in the market for longer period compared to others. Therefore, more evidence is available which could influence the conclusions with regards to their efficacy and safety. A characteristic example is phenobarbital for which, compared to other AEDs, there are more reports of not only its efficacy but also its adverse effects.

As it was found in the previous canine systematic reviews and meta-analysis [8, 15], some characteristics may have also adversely affected the evaluation of the included studies. Similarly, multiple factors could have influenced our results such as signalment differed between studies, heterogeneity in treatment methods among studies, variation in study publication dates, publication bias, several introduced biases detected, lack of high quality evidence studies (i.e. bRCTs and bRELAS), lack of well characterized diagnostic procedures and enrolment of relatively small study population.

### Implications for research

Systematic reviews are a good step towards clinical evidence medicine, however the evaluation and comparison among AEDs through a meta-analysis could provide far further information and aid clinician’s decision to choose the most appropriate AED for every patient. A meta-analysis was not feasible here due to the variations in baseline characteristics of the cats involved, the significant differences between study designs, the several sources of identified bias and mainly due to the lack of comparison group studies. Although the 95% CI and the prevalence of successfully treated cases and adverse effects in each study provide a general indicator of each AED’s efficacy and safety profile, respectively, and can lead to indirect comparisons between AEDs, comparison group studies are essential as they allow thorough statistics to be performed for direct and thus more reliable comparisons. Therefore, further comparison groups and blinded randomized studies are essential for feline epilepsy treatment that would allow a meta-analysis towards this goal.

Lastly, a further problem that was found during the studies evaluation was the lack of reported information. This led to difficulties in performing statistical analysis for AEDs’ comparisons. Therefore, it is essential that future trials should provide precise information and scientists have open access to trials’ data. It would be crucial that journals enforce authors to report their results based on guidelines such as STROBE (Strengthening the Reporting of Observational studies in Epidemiology), ARRIVE (Animal Research: Reporting of In Vivo Experiments) and CONSORT (Consolidated Standards of Reporting Trials).

### Implications for clinical practice

Current evidence did not allow comparisons among AEDs, and therefore it would be rather inaccurate to make definite statements on which one should be considered as a first or second choice in terms of both efficacy and safety profile. However, if clinicians focus on AED’s efficacy, phenobarbital can be used as first-choice monotherapy and if they focus on AED’s safety, imepitoin or levetiracetam can be used. It is important to report that, similarly to canine epilepsy [8], phenobarbital should not be overstated as an AED with high incidence of detrimental adverse effects and therefore, in healthy cats with no pre-existing liver disease, phenobarbital might be the most appropriate choice. Levetiracetam or imepitoin can also be considered as a safe alternative for monotherapy, especially in cats that develop unexpected adverse effects to phenobarbital. For certain epilepsy phenotypes, such as myoclonic epilepsy in elderly cats, levetiracetam monotherapy can be considered. Levetiracetam, imepitoin or phenorbarbital and, to a quite lesser degree, zonisamide might be considered as add-on medication when first line treatment chosen is not sufficient to control seizures. Potassium bromide can be used as adjunctive AEDs only as a last resort and after a signed owner’s consent form in cases manifesting resistance to >2 AEDs and should be closely monitored for type II adverse effects. Diazepam might not be an appropriate choice due to the inadequate evidence supporting its efficacy and the possibility of severe and potentially life-threatening type II adverse effects. It is also important to note that the recommendations made are on the basis of the evidence provided in the available literature and local drug legislation need to be considered prior of prescribing medications.

In general, as it was found in our previous systematic review [8], most of the AED adverse effects documented were inconsistent, fairly tolerable and not life-threatening and ceased once doses and serum levels were decreased or following drug withdrawal. Exceptions included specific type II adverse effects and specific antiepileptic drugs (in particular diazepam and to a lesser degree potassium bromide). It is essential that clinicians assess both the benefits (i.e. value for money, dosing regimen, efficacy) and risks (i.e. safety and tolerability, and impact of adverse effects on the cat’s and owner’s quality of life) before choosing a specific AED.

## Conclusion

This systematic review provides an evidence-based assessment of the data on the AEDs’ efficacy and adverse effects for feline epilepsy. Factors that need to be considered when evaluating these results are: i) drugs that have been used and been on the market for longer periods provided more evidence, ii) the vast majority of the studies offered overall high risk of bias and included small number of cats with unclear or fair disease characterization criteria and short-term follow-up. Individual AED assessments of efficacy and safety profile showed that phenobarbital can be used as the first-choice AED followed by imepitoin and levetiracetam. Further studies in feline epilepsy treatment are by far essential in order to establish definite guidelines for AEDs’ efficacy and safety.

## Additional file

Additional file 1: PRISMA flow diagram. (DOC 56 kb)

Additional file 2: **Table S1**. Details of feline population size, seizure frequency, treatment time, doses of AED(s), seizure frequency reduction after AED initiation, 95% CI for the successful and affected cases and evidence statements for each study. (DOCX 32 kb)

## Abbreviations

AED(s): anti-epileptic drug(s)
BID: bis in die (twice daily)
bELAS: blinded experimental laboratory animal studies
bRCTs: blinded randomised clinical trials
CI: confidence interval
CTs: clinical trials
GI: gastrointestinal
IE: idiopathic epilepsy
LEV: levetiracetam
m: month(s)
MRI: magnetic resonance imaging
NA: Not Available
nbELAS: non-blinded experimental laboratory animal studies
nbRCTs: non-blinded randomised clinical trials
NRELAS: non-randomised experimental laboratory studies
NRCTs: non-randomised clinical trials
PB: phenobarbital
PBr: potassium bromide
PD: polydipsia
PO: per os
PP: polyphagia
PU: polyuria
SID: semel in die (once daily)
TID: ter in die (three times daily)
UCTs: uncontrolled clinical trials
UELAS: uncontrolled experimental laboratory studies
w: week(s)
