# Supplementary material for: Systematic review of antiepileptic drugs’ safety and effectiveness in feline epilepsy
Source: BMC Vet Res. 2018 Mar 2;14:64. doi: 10.1186/s12917-018-1386-3 (PMC5834883; doi:10.1186/s12917-018-1386-3)
Supplement: Supplementary file 1 — PRISMA flow diagram. (DOC 56 kb) [file 12917_2018_1386_MOESM1_ESM.doc]

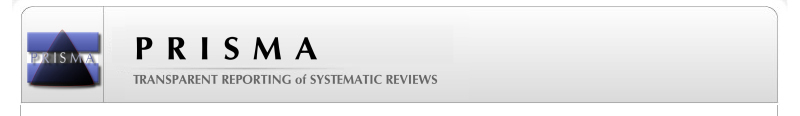
**PRISMA Flow Diagram**

**Screening**

**Included**

**Eligibility**

**Identification**

Records identified through database searching
(n = 679 )

Additional records identified through other sources
(n = 5 )

Records after duplicates removed
(n = 248 )

Records screened
(n = 248 )

Records excluded
(n = 76 )

Full-text articles assessed for eligibility
(n = 176 )

Full-text articles excluded, with reasons
(n = 136 )

Studies included in qualitative synthesis
(n = 40 )

Studies included in quantitative synthesis (meta-analysis)
(n = 0 )
