# Supplementary material for: Systematic review of antiepileptic drugs’ safety and effectiveness in feline epilepsy
Source: BMC Vet Res. 2018 Mar 2;14:64. doi: 10.1186/s12917-018-1386-3 (PMC5834883; doi:10.1186/s12917-018-1386-3)
Supplement: Supplementary file 2 — Table S1. Details of feline population size, seizure frequency, treatment time, doses of AED(s), seizure frequency reduction after AED initiation, 95% CI for the successful and affected cases and evidence statements for each study. (DOCX 32 kb) [file 12917_2018_1386_MOESM2_ESM.docx]

Table 2 Details of feline population size, seizure frequency, treatment time, doses of AED(s), seizure frequency reduction after AED initiation, 95% CI for the successful and affected cases and evidence statements for each study

| References | Pakozdy et al. [27] | Baho et al. [22] | Finnerty et al. [25] | Solomon et al. [34] | Ducote et al. [23] | Boydell [29] | Lowrie et al. [26] | Volk et al. [28] | Cochrane et al. [32] | | Cochrane et al. [33] | Cuff et al. [30] | Lieser and Schwedes [24] | Schriefl et al. [4] | | Gasper et al [35] | Wahle et al. [31] |
| --- | --- | --- | --- | --- | --- | --- | --- | --- | --- | --- | --- | --- | --- | --- | --- | --- | --- |
| AED evaluated | Phenobarbital | | | | | | | | | | | | | | | | |
| 2^nd^ AED | Diazepam (3) | - | - | - | - | - | - | - | | - | - | LEV | - | - | - | |  |
| 3^rd^ AED | Gabapentin (2) | - | - | - | - | - | - | - | | - | - | - | - | - | - | |  |
| 4^th^ AED | LEV (1) | - | - | - | - | - | - | - | | - | - | - | - | - | - | |  |
| No of cats | 36 | 1 | 19 | 6 | 1 | 1 | 34 (29 completed the efficacy analysis) | 16 | | 8 (non-epileptic cats) | 7 (non-epileptic cats) | 1 | 1 | 17 | 19 | | 18 |
| Age of cats at seizure onset (years) | mean 4.81; range 0.5-11 | 4.5 | median 6; range, 0.75-18 years (cats with IE) and 2.5; range, 2-7 (cats with presumed IE) | NA | 1.5 | 4 | median 16; range 10-19 | NA | | range 1-3 | range 1-2 | 10 | 2 | median 3, mean 3.54, range 1-12 | NA | | median 3.8; range 0.4-14.4 |
| Period of treatment or follow-up (months) | mean 50; range 13-158 | 1 | median 8; range, 0.63-72.3 (cats with IE) and median 75.8; range, 27.3-91 (cats with presumed IE) | 0.75 | 1 | 14 | 3 | NA | | 0.75 | 0.75 | 2 | 1 | median 37, mean 32, range 0.5-71 | 0.5 | | median 19.2; range, 12–75.6 |
| Dose of AED(s) (mg/kg) | PB: mean 1.6; range 0.9-5 PO BID; LEV: 16 PO TID; gabapentin: 16 PO TID | 2.7 PO BID | median 2; range 1-3.8 PO BID (cats with IE) and median 2.15; range, 1.4-3.25 PO BID (cats with presumed IE) | 10-20 intraperi-toneally SID | 4 PO BID | 1 PO BID | PB: median 3.12; range 1.67-7.5 PO BID | NA | | 10 mg/kg intravenously and PO (one single dose per route, 3 weeks apart) | 5 PO SID | PB: 4.5 PO BID; LEV: 50 PO TID | 2.3 PO BID | NA | 3 or 9 transdermal BID | | range, 1.3–4.4 PO BID |
| Serum levels of AED(s) | mean 27.9; range 12–52 μg/ml (seizure-free cats) and mean 31.2; range 11–73 μg/ml (remaining cats) | 27.5 μg/ml | median 29; range 8-44 μg/ml (cats with IE) and median 28; range, 26-31 μg/ml (cats with presumed IE) | 12–52 μg/ml | 39.1 μg/ml | NA | mean 27.7; range 20.4-33.2 μg/ml | NA | | 13 μg/ml (1 day after administration) | mean 16.4; range 13.4-19.3 μg/ml | NA | 25.7 mg/l | NA | 0-37 μg/ml | | range, 12.7-45.4 mg/ml |
| Pre-treatment SF (seizures/month or year) | range 2 - >10 / year (recorded over a period of year) | approximately 3/year (recorder over a period of 6-12 months) | mean 1.71/month (recorded over a period of 9 m) | NA | NA | approximately 30/month | median 70/month | NA | | NA | NA | continuous | NA | NA | NA | | NA |
| Post-treatment SF (seizures/month or year) | range 0 - >10 / year (recorded over a period of approximately 10 years) | NA | mean 0.59 | NA | NA | Approximately 8/month | NA | NA | | NA | NA | 0 (recorded over a period of 2 motnhs) | NA | NA | NA | | NA |
| No of cats that were failures | 6/36 (16%) | NA | - | NA | NA | - | NA | - | | NA | NA | - | NA | NA | NA | | 2/18 (11%) |
| No of cats with >0% - <50% reduction in SF | 4/36 (10%) | NA | 1/19 (5%) | NA | NA | - | 28//29 (97%) | 4/16 (25%) | | NA | NA | - | NA | NA | NA | | NA |
| No of cats with ≥50% - <100% reduction in SF | 11/36 (31%) | NA | 18/19 (95%) | NA | NA | 1/1 (100%) | 1/29 (3%) | 5/16 (31%) | | NA | NA | - | NA | NA | NA | | 3/18 (17%) |
| No of cats with 100% reduction in SF | 15/36 (43%) | NA | NA | NA | NA | - | - | 7/16 (44%) | | NA | NA | 1/1 (100%) | NA | 10/17 (59%) | NA | | 8/18 (44%) |
| 95% CI of successfully treated cases | 56% - 84% | NA | 75%-99% | NA | NA | 100% | 0.16-17% | 50%-90% | | NA | NA | 100% | NA | 36-78% | NA | | 39-80% |
| Prevalence of adverse effects | 26/36 (72%) | NA | 0% | 6/6 (100%) | NA | NA | 11/34 (32%) | NA | | 0% | 0% | NA | NA | NA | 13/19 (68%) | | 1/18 |
| 95% CI of cases that developed adverse effects | 56-84% | NA | 0% | 100% | NA | NA | 19-49% | NA | | 0% | 0% | NA | NA | NA | 46%-85% | | 0.9-26% |
| Body system affected and adverse effects | Neurological (sedation(15), ataxia(9), paraparesis(6), behavioral changes(1)), GI (weight loss(3), PP(6)), PD(1), Dermatological (dermatitis(2), pruritus(2)) ClinPath (increased ALT(11), ALP(4), leucopenia(4), thrombocytopenia(1)) | Lymphoreticular (pseudolymphoma) | NA | ClinPath (reduction in Vitamin K-dependent clotting factors) | Lymphoreticular (enlarged lymph nodes), GI (multiple ulcerative and exudative oral lesions), dermatological (ulcerative dermatitis on pinna) | NA | Neurological (sedation (8), ataxia (4), behavioural changes (1)) | Neurological (sedation), PP | | NA | NA | NA | Lymphoreticular (pseudolymphoma) | NA | Neurological (sedation (2), ataxia (3), paraparesis (2)) (GI (PP(3)), PU(1), PD (2) | | Clinpath (leucopenia) |
| Most common adverse effects | Sedation, ataxia, paraparesis, increased ALT | NA | NA | NA | NA | NA | Sedation | NA | | NA | NA | NA | NA | NA | PP | | NA |
| Adverse effect type | I & II | II | NA | II | II | NA | I | I | | NA | NA | NA | II | NA | I | | II |
| Proportion of specific adverse effects for each AED based on all study reports | Type I: sedation (4/12; 33%), ataxia (3/12; 25%) paraparesis (2/12; 17%), behavioral changes (2/12; 17%), dermatitis (2/12; 17%), pruritus (1/12; 8%), elevated serum ALP (1/12; 8%) and ALT (1/12; 8%), PP (3/12; 25%), PD (2/12; 17%) and weight loss (1/12; 8%) | | | | | | | | | | | | | | | | |
|  | Type II: leucopenia (2/12; 17%), thrombocytopenia (1/12; 8%) and coagulopathy (1/12; 8%), pseudolymphoma (3/12; 25%), ulcerative stomatitis (1/12; 8%) | | | | | | | | | | | | | | | | |
| Proportion of specific adverse effects for each AED based on the total affected population | Sedation (33/147; 22%), PP (17/147; 12%), ataxia (16/147; 11%), elevated serum ALT (11/147; 7%), paraparesis (8/147; 5%), elevated serum ALP (4/147; 3%), leucopenia (5/147; 3%), weight loss (3/147; 2%), dermatitis (3/147; 2%), pseudolymphoma (3/147; 2%), PD (3/147; 2%), behavioral changes (2/147; 1%), pruritus (2/147; 1%), thrombocytopenia (1/147; <1%), ulcerative stomatitis (1/147; <1%) and vitamin-K dependent clotting factors reduction (1/147; <1%) | | | | | | | | | | | | | | | | |
| Overall level of evidence supporting the efficacy and safety profile of an AED | Weak level of evidence for phenobarbital’s efficacy and safety profile | | | | | | | | | | | | | | | | |

*AED(s)* anti-epileptic drug(s), *BID* bis in die (twice daily), *CI* confidence interval, *GI* gastrointestinal, *IE* idiopathic epilepsy, *LEV* Levetiracetam, *m* month(s), *NA* Not Available, *PB* phenobarbital, *PD* polydipsia, *PU* polyuria, *PP* polyphagia, *PBr* potassium bromide, *PO* per os, *SID* semel in die (once daily), *TID* ter in die (three times daily), *w* week(s), *y* year(s)
